# Illuminating Hope for Mental Health: A Drug Review on Lumateperone

**DOI:** 10.7759/cureus.46143

**Published:** 2023-09-28

**Authors:** Martin Tarzian, Mariana Ndrio, Byron Chique, Japjit Serai, Bryce Thalackal, Jessi Lau, Adegbenro O Fakoya

**Affiliations:** 1 Psychiatry, University of Medicine and Health Sciences, Basseterre, KNA; 2 Psychiatry and Behavioral Sciences, University of Medicine and Health Sciences, Basseterre, KNA; 3 Surgery, University of Medicine and Health Sciences, Basseterre, KNA; 4 Medicine, University of Medicine and Health Sciences, Basseterre, KNA; 5 Human Health, University of Guelph, Guelph, CAN; 6 Cellular Biology and Anatomy, Louisiana State University Health Sciences Center, Shreveport, USA

**Keywords:** drug review, schizophrenia, bipolar disorder, caplyta, lumateperone

## Abstract

This drug review provides a comprehensive analysis of a novel antipsychotic called lumateperone, marketed as Caplyta. Lumateperone gained FDA approval in 2019 for treating schizophrenia and later, in 2021, for treating bipolar depression. The review begins by delving into lumateperone's mechanism of action, which involves the partial agonism of the dopamine D2 receptor as well as its unique effects on the dopamine transporter, N-methyl-D-aspartate (NMDA) receptor, and serotonin transporter. Additionally, the study examines lumateperone's distinctive pharmacokinetics. Moreover, this review assesses lumateperone's metabolic profile and highlights its favorable outcomes regarding mean body weight, BMI, and waist circumference, surpassing those of other second-generation antipsychotic medications. The study explicitly emphasizes the efficacy and safety of lumateperone in treating schizophrenia and bipolar depression associated with bipolar I and II disorders. An extensive investigation of multiple clinical trials provides compelling evidence of lumateperone's advantages over existing antipsychotic medications. The review also acknowledges the limitations of lumateperone compared to other antipsychotics. In conclusion, this drug review underscores the importance of further research to uncover the additional limitations of lumateperone while acknowledging its promising benefits and potential for advancing treatment options.

## Introduction and background

The treatment of mental illness is fast-paced and ever-evolving. Whenever a new psychotropic drug makes it to the market, new hope of relief for those who suffer from mental illnesses is offered. Mental illness is a burden that causes diminished quality of life, comorbidity, and decreased life expectancy [1]. The demand for secure and reliable pharmacological therapies increases daily as mental health issues rise globally.

Promising advancements in pharmacological treatments have brought optimism to the field of psychiatry. Lumateperone, marketed under the brand name Caplyta (Intra-Cellular Therapies, Inc., New York, NY, United States), is one of the most recent additions to the current arsenal of psychotropic medications [2]. Lumateperone’s unique mechanism of action, involving modulation of serotonin, dopamine, and glutamatergic neurotransmission, has garnered considerable attention for its potential effectiveness in treating schizophrenia and bipolar depression [2]. Its unique mechanism, coupled with its favorable side effect profile, led to lumateperone receiving FDA approval in 2019 for treating adult patients with schizophrenia [3,4]. Furthermore, in 2021, the FDA expanded its approval to include lumateperone as monotherapy as well as an adjunctive treatment for bipolar depression associated with both bipolar I and II disorders [5]. With this expanded indication, lumateperone became one of only four treatments approved by the FDA for both bipolar I and bipolar II depression [5].

This review provides a thorough analysis of lumateperone’s unique mechanism of action and its efficacy in treating various neuropsychiatric disorders such as schizophrenia and bipolar disorders. The review focuses on evaluating the outcomes of clinical studies while also discussing the latest research analyses and ongoing clinical trials. Furthermore, it summarizes the safety and tolerability data from multiple studies, assessing treatment-emergent adverse events and lumateperone's metabolic profile. The aim is to present a comprehensive profile of lumateperone that will serve as a valuable resource for clinicians and researchers, offering insights into its therapeutic potential and its use in clinical practice.

## Review

The mechanism of action of first- and second-generation antipsychotics

Antipsychotics, both typical and atypical, normally antagonize dopamine transmission at the dopamine D2 receptor subtype, although a few second-generation antipsychotics (SGAs) exhibit partial agonist activity. This mechanism of action is theorized to play a pivotal role in their therapeutic properties [1]. First-generation antipsychotics (FGAs) with potent D2-blocking properties were associated with side effects known as extrapyramidal syndrome (EPS) [1]. EPS is a constellation of side effects, including acute dystonia (involuntary contractions of the head and neck), akathisia (the inability to sit still), tardive dyskinesia (involuntary movements of the face), and drug-induced Parkinson's disease [1]. Clozapine, an SGA, has demonstrated more user-friendly antipsychotic action without significant EPS [1]. Subsequent SGAs aimed to mimic these properties by combining potent antagonism at the serotonergic 5-hydroxytryptamine (serotonin) type 2A (5-HT2A) receptor with weaker D2 antagonism [1]. 

The reduced risk of EPS in SGAs is attributed to their higher affinity for the 5-HT2A serotonergic receptor than the D2 receptor. Clozapine, in particular, has an almost negligible risk of causing EPS. It is not surprising that clozapine also has one of the lowest affinities for D2 receptors and the highest affinities for 5-HT2A serotonergic receptors of all the SGAs [6]. However, it is essential to note that clozapine is associated with other serious risks, such as agranulocytosis, ileus, seizures, and marked sedation [6]. Thus, clozapine is only used when other treatment modalities have failed. Risperidone, quetiapine, and olanzapine also demonstrate fewer extrapyramidal side effects than their FGA counterparts. Risperidone, however, poses the greatest risk of causing EPS of all the SGAs [6]. Despite the proven effectiveness of quetiapine and olanzapine in treating both schizophrenia and bipolar disorder [1], they have their own set of metabolic side effects, which will be discussed in “The metabolic profile of lumateperone” section of this review.

Although the mechanism of action of both FGAs and SGAs is effective in managing schizophrenia and bipolar disorder, they are far from perfect. They are known to have side effects, and in some cases, treatment resistance has been noted [6]. Although the side effect profile of SGAs is much more tolerable than that of the FGAs, they still occur [1,5]. In this regard, the newer generation antipsychotics, such as lumateperone, have novel mechanisms of action that may be advantageous for treating these patient populations, with even fewer EPS and metabolic side effects.

Lumateperone’s unique mechanisms of action

**Figure 1:**
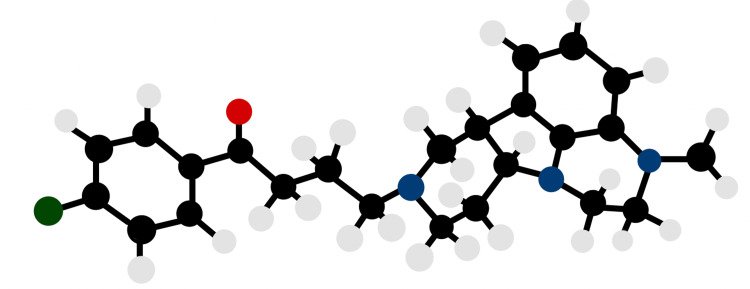
The chemical structure of lumateperone. The chemical name of lumateperone is 4-((6bR,10aS)-3-methyl-2,3,6b,9,10,10a-hexahydro-1H,7H-pyrido[3’,4’:4,5]pyrrolo[1,2,3-de]quinoxalin8-yl)-1-(4-fluoro-phenyl)-butan-1-one 4-methylbenzenesulfonate [4]. Black circles represent carbon atoms, white circles represent hydrogen atoms, blue circles represent nitrogen atoms, green circle represents a fluoride atom, and red circle represents an oxygen atom. Image credit: Jessi Lau.

Lumateperone demonstrates a higher affinity for dopamine D2 receptors, particularly in mesolimbic and mesocortical regions, in contrast to other SGAs [7]. Notably, its unique pharmacological activity as a presynaptic partial agonist at dopamine D2 receptors sets it apart from traditional SGAs [7]. Lumateperone has robust and dose-dependent partial agonist activity at dopamine D2 receptors that may normalize dopamine transmission and reduce the risk of excessive dopamine blockade-related side effects, such as EPS [7,8]. In the various trials discussed in this review, lumateperone shows little to no EPS side effects. The chemical structure of lumateperone is shown in Figure 1.

**Figure 2:**
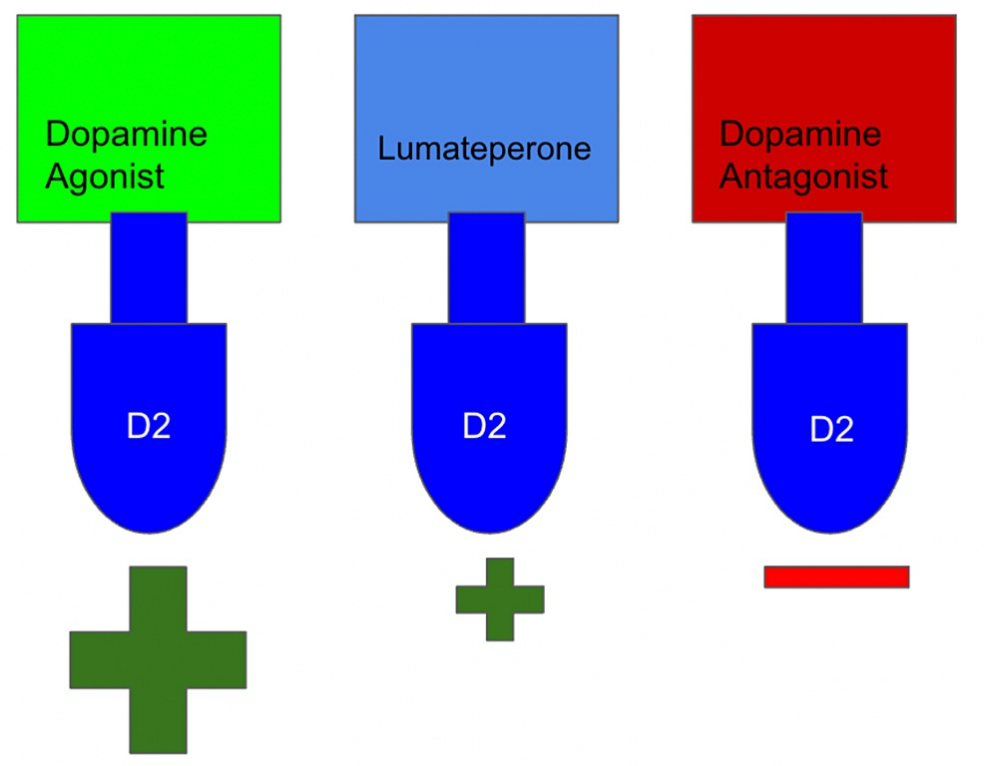
Comparative effects of dopamine agonists, lumateperone, and antagonists on dopaminergic activity. This figure illustrates the varying levels of dopaminergic activity induced by different classes of drugs. Dopamine agonists exhibit robust stimulation of dopaminergic receptors, leading to increased activity. Lumateperone, a partial agonist, demonstrates mild dopaminergic activity, striking a balance between stimulation and inhibition. Antagonists, on the other hand, effectively block dopaminergic activity by binding to receptors and preventing their activation. Understanding these distinct mechanisms can inform drug development and treatment strategies for dopaminergic disorders. Image credit: Martin Tarzian

Aripiprazole, one of the earlier antipsychotic medications with partial dopamine agonist activity, has demonstrated a lower incidence of EPS than most other antipsychotics [1,9]. The rationale behind this observation is that the partial agonist activity of aripiprazole allows for a more balanced modulation of dopamine receptors, reducing the risk of excessive dopamine blockade and subsequent EPS [9]. In this regard, it is reasonable to speculate that lumateperone, which also possesses partial agonist activity at dopamine receptors, may have a lower EPS profile as well [1,9]. A similar mechanism of action suggests that lumateperone may also offer a balanced modulation of dopamine transmission, minimizing the risk of EPS commonly associated with traditional antipsychotics that act solely as dopamine receptor antagonists [1,9,10]. Figure 2 illustrates the impact of various agents on dopaminergic levels.

**Figure 3:**
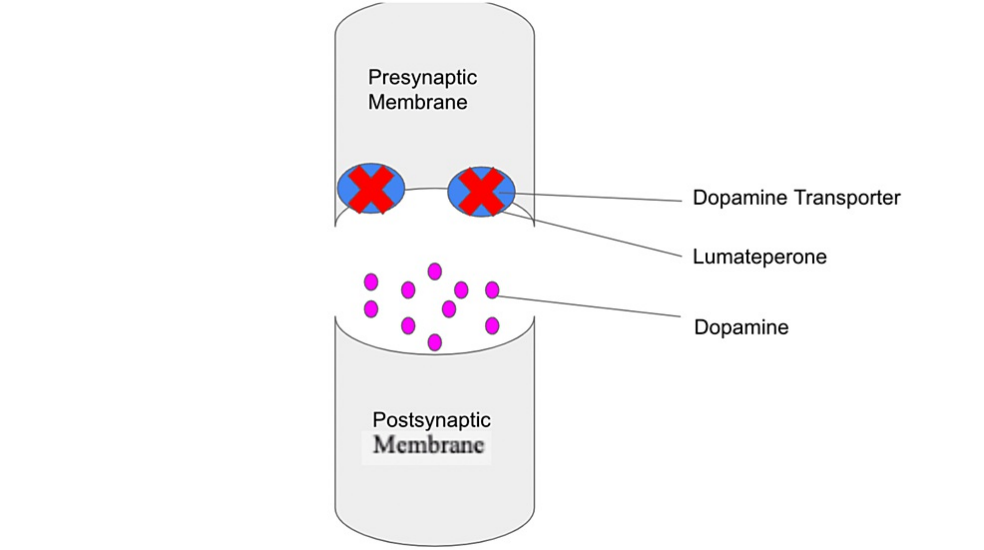
Lumateperone's mechanism of action: blocking DATs and increasing dopamine in the synaptic cleft. This figure depicts lumateperone's unique mechanism of action, which blocks DAT responsible for the reuptake of dopamine from the synaptic cleft. By inhibiting DAT, lumateperone accumulates dopamine in the synaptic cleft, thereby increasing dopaminergic signaling. This novel mode of action holds potential implications for treating dopamine-related disorders and provides insight into lumateperone's therapeutic effects. DATs: dopamine transporters. Image credit: Martin Tarzian

The inhibition of the dopamine transporter (DAT) is a unique characteristic of lumateperone that sets it apart from other antipsychotic medications and may contribute to its enhanced effectiveness in treating schizophrenia [10]. By inhibiting the DAT, lumateperone prevents dopamine reuptake from the synaptic cleft, increasing dopamine levels in the mesocortical pathway, which is a typical hypofunction in schizophrenia [10], as shown in Figure 3. This mechanism of action helps to modulate dopamine neurotransmission and restore the balance of dopamine signaling, which is often severely antagonized by other antipsychotics [10]. Similar to its partial activity at dopamine receptors, this unique property of lumateperone may contribute to its lack of EPS symptomatology [10]. 

**Figure 4:**
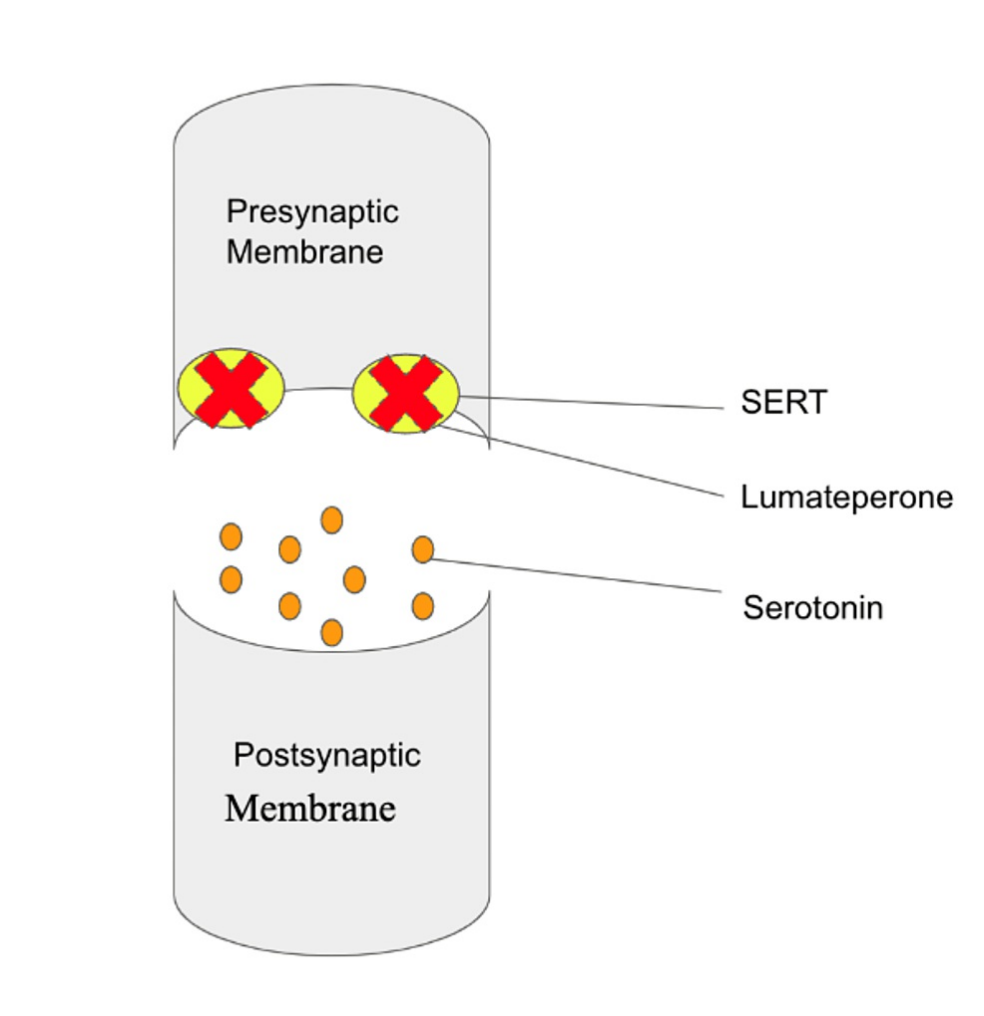
Lumateperone's mechanism of action: blocking SERT and modulating serotonin in the synaptic cleft. This figure illustrates lumateperone's distinct mechanism of action by blocking SERT responsible for serotonin reuptake from the synaptic cleft. By inhibiting SERT, lumateperone hinders the reabsorption of serotonin, leading to increased serotonin levels in the synaptic cleft. This modulation of serotonin signaling offers potential therapeutic benefits for serotonin-related conditions, highlighting lumateperone's multifaceted pharmacological effects. SERT: serotonin transporter. Image credit: Martin Tarzian

Lumateperone exhibits yet other novel mechanisms of action in the treatment of schizophrenia. By acting as an agonist at D1 receptors, lumateperone enhances the activation of N-methyl-D-aspartate (NMDA) receptors, leading to increased phosphorylation [9]. In individuals with schizophrenia, impaired NMDA-mediated glutamate signaling has been observed [9]. Lumateperone addresses this by promoting phosphorylation and facilitating enhanced glutamate signaling [9]. Furthermore, lumateperone exerts inhibitory effects on the serotonin transporter (SERT), increasing serotonin levels in the synaptic cleft as shown in Figure 4. This modulation of serotonin levels can have significant implications for treating schizophrenia and bipolar depression, as serotonin is involved in various neurochemical pathways and plays a role in mood regulation, cognition, and overall brain function [9].

The dual actions of lumateperone, increasing phosphorylation at NMDA receptors to enhance glutamate signaling and inhibiting SERT to augment serotonin levels, highlight its unique pharmacological profile. Coupled with its ability to modulate dopaminergic transmission better than any other psychotropic drug, lumateperone offers a novel approach to address the neurochemical imbalances associated with schizophrenia. These distinct mechanisms of action set lumateperone apart from conventional antipsychotics and suggest its potential as an innovative therapeutic option for individuals with schizophrenia and bipolar disorder. Other pharmacodynamic properties of lumateperone include a moderate affinity for dopamine D4, α-1A, and α-1B adrenergic receptors, while displaying a low affinity for muscarinic and histaminergic receptors [7]. As discussed in "The metabolic profile of lumateperone" section, the muscarinic and histaminergic properties of lumateperone may explain its decreased risk for metabolic syndrome.

The pharmacokinetics of lumateperone

Lumateperone exhibits specific pharmacokinetic properties. Upon oral administration, lumateperone is absorbed and reaches a maximum concentration (*T*_max_) within three to four hours [11]. Plasma concentrations of lumateperone and its metabolites can be detected up to eight hours after administration [11]. The bioavailability of lumateperone is approximately 4.4%, with peak plasma concentration achieved within one to two hours [4]. Steady-state concentrations are reached after five days of multiple doses [4].

The distribution of lumateperone in the body shows a volume of distribution of approximately 4.1 L/kg. It has a high binding affinity to plasma proteins, with 97.4% of the compound bound at therapeutic concentrations [4]. Lumateperone undergoes extensive metabolism by various enzymes, producing more than 20 metabolites [4]. Regarding excretion, lumateperone and its glucuronidated metabolites are primarily excreted in the urine (58%), with a small percentage (less than 1%) excreted unchanged. The terminal half-life of lumateperone is approximately 18 hours, and its clearance is about 17.9 L/h [4].

The pharmacokinetics of lumateperone are generally not significantly influenced by factors such as age, sex, or race. However, co-administration with moderate or potent CYP3A4 and UGT inhibitors should be avoided, as it may increase the risk of toxicity [7]. Additionally, co-administration with compounds that induce or inhibit CYP3A4 is contraindicated due to the potential for increased lumateperone concentrations [4]. The high plasma protein binding of lumateperone suggests that the compound may have a prolonged duration of action in the body [12,13]. Currently, lumateperone is administered orally in the form of capsules, which contain the crystalline tosylate salt formulation [7].

Overall, understanding the pharmacokinetic characteristics of lumateperone provides insights into its absorption, distribution, metabolism, and excretion, contributing to a comprehensive understanding of its therapeutic profile.

The CATIE trial and metabolic effects of current SGAs

The Clinical Antipsychotic Trials of Intervention Effectiveness (CATIE) trial was a landmark study that compared the effectiveness and side effects of various antipsychotic medications, including both FGAs and SGAs [13]. The CATIE trial shed light on the side effects of SGAs in real-world settings, highlighting the serious side effects associated with SGAs [13]. Participants taking SGAs, such as olanzapine and clozapine, experienced significant weight gain compared to those taking FGAs. This weight gain led to long-term metabolic complications, including dyslipidemia and hyperglycemia [13].

Another side effect of SGAs highlighted by the CATIE trial was the increased risk of metabolic syndrome [13]. Metabolic syndrome is a constellation of conditions, including obesity, high blood pressure, abnormal lipid levels, and insulin resistance [13]. SGAs, particularly olanzapine and clozapine, were more strongly associated with metabolic syndrome than their FGA counterparts [13]. The CATIE trial also highlighted the risk of EPS associated with both FGAs and SGAs. The trial found that FGAs were more likely to cause EPS, while SGAs, particularly risperidone, carried a higher risk of causing akathisia [13]. Overall, the CATIE trial provided valuable insights into the side effects of SGAs and contributed to the understanding of their relative effectiveness compared to FGAs. It emphasized the importance of considering the therapeutic benefits and potential adverse effects when selecting an antipsychotic medication for individuals with schizophrenia [13].

The metabolic profile of lumateperone

The analysis of two randomized, placebo-controlled studies (Study 401 and Study 404) evaluated the metabolic profile of lumateperone 42 mg monotherapy in patients with a major depressive episode associated with bipolar I or bipolar II disorder [14]. A post hoc pooled analysis compared the rates of metabolic syndrome between lumateperone and placebo in the treatment of bipolar depression [14]. The study included 746 patients (372 on lumateperone and 374 on placebo). The rates of metabolic syndrome were similar between the two groups at baseline (lumateperone: 20.7%; placebo: 22.2%) and at the end of treatment (lumateperone: 21.8%; placebo: 23.8%) [14]. Interestingly, a higher percentage of patients on lumateperone (36.4%) improved from having metabolic syndrome at baseline to no longer meeting the criteria at the end of treatment compared to placebo (30.1%) [14]. The rate of new-onset metabolic syndrome during treatment was similar between lumateperone (10.8%) and placebo (10.7%) [14]. Based on this post hoc analysis, lumateperone 42 mg demonstrated similar rates of metabolic syndrome compared to placebo in treating bipolar depression. These findings suggest that lumateperone has a promising and favorable metabolic profile [14].

Lumateperone does not have metabolic side effects due to its low affinity for several receptor targets associated with metabolic and cardiovascular complications caused by other antipsychotic treatments [15]. It has a low binding affinity to the 5-HT2C subtype of the serotonin receptor, α1-adrenergic receptor, H1 histaminergic receptor, and muscarinic receptors [15]. These receptors have been linked to disrupted metabolic profiles, leading to obesity and type II diabetes in SGAs [13]. SGAs, such as olanzapine, have potent effects on these receptors, resulting in significant weight gain and elevated plasma cholesterol, glucose, and free fatty acids [13]. Additionally, the cardiovascular effects of other antipsychotic agents, including H1 histaminergic and α-adrenergic receptor effects, are associated with cardiac liabilities and various side effects [15]. In comparison, lumateperone has a consistently low affinity for these off-target receptors compared to most SGA drugs, suggesting a reduced risk of metabolic and cardiovascular complications [15].

In the long term, lumateperone does not have metabolic side effects, as evidenced by a study evaluating its impact on weight and metabolic parameters in patients with stable schizophrenia [16]. The study included patients who were switched from standard-of-care (SOC) treatment to lumateperone 42 mg for up to one year. The findings of the study demonstrated the favorable metabolic effects of lumateperone. Mean cholesterol levels (total and low-density lipoprotein (LDL)) significantly decreased from those of SOC treatment [16]. Moreover, there were significant improvements in mean body weight, BMI, and waist circumference during the one-year treatment with lumateperone. Notably, potentially clinically significant weight loss occurred in 19% of the population, including obese and overweight patients. Conversely, weight gain was infrequent [16].

**Figure 5:**
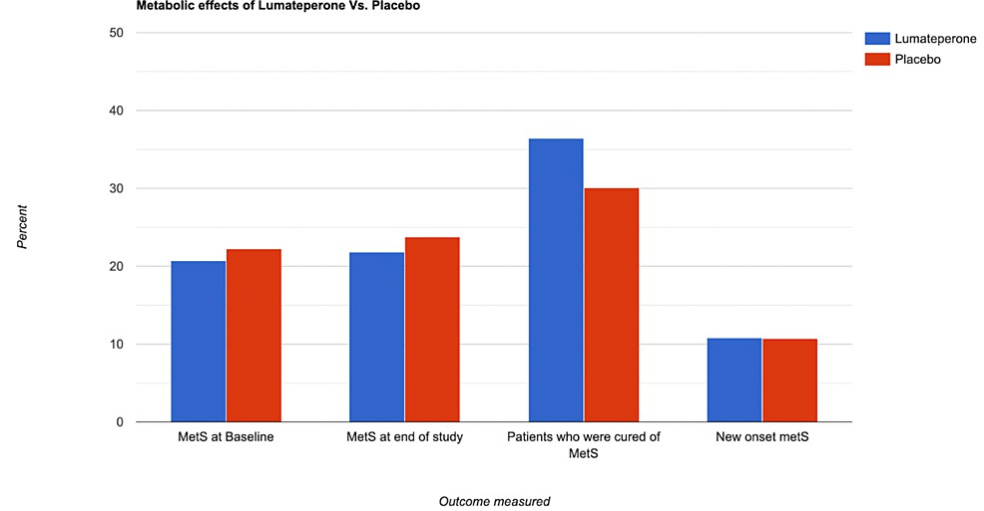
The results of Study 401 and Study 404. No significant difference in MetS between placebo and lumateperone. MetS: metabolic syndrome. Image credit: Martin Tarzian, made with Rapidtables.com.

The study also revealed positive shifts in BMI categories, with 28% of patients transitioning from overweight to normal BMI and 21% improving from obese to overweight [16]. These results suggest that lumateperone 42 mg has the potential to be a promising new treatment for schizophrenia with minimal metabolic risk [16]. Overall, the long-term use of lumateperone in patients with stable schizophrenia showed improvements in metabolic and weight parameters, particularly in patients who were overweight or obese at the beginning of the study [16]. These findings support the notion that lumateperone has a favorable metabolic side effect profile compared to SOC treatments for schizophrenia, potentially reducing patient morbidity and improving outcomes [16]. The findings of these studies are demonstrated in Figure 5.

Lumateperone in the treatment of schizophrenia: past and present clinical studies

Lumateperone's unique mechanism of action makes it a promising candidate for joining other antipsychotics as a first-line option for treating and managing schizophrenia. During its development, Intra-Cellular Therapies, Inc. conducted clinical studies that demonstrated the efficacy and safety of lumateperone in treating schizophrenia. This led to the FDA approval of lumateperone for use in adult patients with schizophrenia in 2019 [9]. This section covers the clinical studies that preceded FDA approval of lumateperone for schizophrenia and discusses new clinical studies currently conducted to evaluate the safety and efficacy of lumateperone in other age populations, doses, and routes of administration.

Exploring the promise: an early drug trial on lumateperone

In 2011, one of the earliest clinical studies (NCT01499563) evaluating the safety and efficacy of lumateperone in treating schizophrenia was conducted, and the results were published by Lieberman et al. in 2016 [17]. This phase II trial was a randomized, double-blind, placebo-controlled study that involved 335 participants across eight sites in the United States diagnosed with schizophrenia or experiencing acute psychosis. Participants were randomized to receive either 42 mg or 84 mg of lumateperone, placebo, or risperidone as monotherapy for four weeks, with the primary outcome measure being the Positive and Negative Syndrome Scale (PANSS) total score [17].

The final results showed that lumateperone 42 mg demonstrated greater antipsychotic efficacy compared to placebo on the primary endpoint (−13.2 points vs. −7.4 points; p = 0.017, effect size = 0.4) significantly reducing positive symptoms relative to the placebo group on the PANSS subscales. On the other hand, while lumateperone 84 mg reduced positive symptoms and general psychopathology, it did not reach statistical significance compared to the placebo group (−8.3 vs. −7.4; p = 0.708). While lumateperone 42 mg and risperidone improved positive symptoms and general psychopathology, only lumateperone 42 mg showed a slight improvement in negative symptoms. However, the improvement did not reach statistical significance, which the investigators attributed to the low baseline levels of negative symptomatology in the patient population. The 84 mg dose of lumateperone did not result in any improvement in negative symptoms at all [17].

Both doses of lumateperone were well tolerated by the patient population, with low discontinuation and adverse events. The incidence of treatment-emergent adverse events (TEAEs) with lumateperone 42 mg was not significantly different from placebo (relative risk = 1.14, p = 0.346), while the relative risk for lumateperone 84 mg was 1.30 (p = 0.024) and for risperidone was 1.25 (p = 0.077). Somnolence/sedation was the most commonly reported TEAE, with higher incidence rates observed with lumateperone 84 mg (32.5%) than with placebo (13%), lumateperone 42 mg (17%), and risperidone (21%). Most importantly, neither dose of lumateperone was associated with EPS [17].

Lumateperone also demonstrated a benign metabolic profile, as evidenced by significantly lower prolactin levels, fasting glucose, total cholesterol, and triglycerides than risperidone [17]. Weight changes from lumateperone varied site-to-site, and compared to risperidone, there was a tendency for less weight gain in the lumateperone groups (p = 0.087 for lumateperone 60 mg vs. risperidone, and p = 0.074 for lumateperone 120 mg vs. risperidone) [17].

Lumateperone for management of acute psychosis

After the study by Lieberman et al., a phase III randomized, double-blind, placebo-controlled trial (NCT02282761) was conducted in 2014, with the results published in 2020 by Correll et al. [18]. The study involved 450 adult patients with schizophrenia experiencing an acute exacerbation of psychosis. They were randomly assigned to receive lumateperone 42 mg, 28 mg, or a placebo once daily for four weeks [18]. This study aimed to investigate if a lower dose of 28 mg lumateperone would show significant improvements in symptoms compared to the lumateperone 42 mg and placebo, given that Lieberman found the higher dose of lumateperone 82 mg did not provide any significant symptom improvements compared to placebo [18]. 

The final analysis showed that the administration of lumateperone 42 mg resulted in a significant improvement in the PANSS total score compared to the placebo (-4.2; p = 0.02). Patients who received lumateperone 28 mg also showed some improvement from baseline but had a minor reduction in symptoms (-2.6; p = 0.16) compared to those who received lumateperone 42 mg, and overall, the 28 mg dose did not differ significantly from placebo [18].

This study further showed that lumateperone was generally well tolerated, with no treatment-emergent motor, metabolic, or endocrine adverse effects compared to the placebo, consistent with the earlier trial [18].

Optimal dosing of lumateperone based on the studies of Lieberman and Correll

In summary, these two major clinical trials demonstrated that lumateperone 42 mg had antipsychotic efficacy in reducing positive and negative symptoms and was well tolerated by patients. However, the higher dose of lumateperone 84 mg did not show significant improvements compared to the placebo in the first study [17], and the lower dose of lumateperone 28 mg, in the second study, was not found to be as effective as the 42 mg dose but was still well tolerated [18]. Overall, these studies contributed to our understanding of lumateperone's therapeutic potential and its favorable safety and metabolic profile compared to other antipsychotic medications in treating schizophrenia. 

Ongoing trials for long-term management of schizophrenia

While additional studies are needed to fully understand the long-term efficacy and safety of lumateperone in the treatment of schizophrenia, ongoing clinical trials are currently evaluating the safety and tolerability of lumateperone in various age populations with different formulations for the treatment of schizophrenia.

One such trial (NCT04709224) was specifically focused on evaluating the safety and tolerability of lumateperone in a long-acting injectable (LAI) form as a treatment option for adult patients with schizophrenia [19]. This study was a phase 1, open-label, non-randomized trial that included 37 adult participants diagnosed with schizophrenia. Before enrolling, these participants had been stable without experiencing a psychotic episode and on a consistent dose of antipsychotic medication, including lumateperone, for at least three months [19]. The participants were divided into four experimental cohorts, each receiving a different dose of lumateperone LAI ranging from 50 to 200 mg. The LAI was administered subcutaneously in the abdomen for most participants, except for those in the last cohort who received it in the outer area of the upper arm. Before administering the LAI lumateperone, all participants received oral lumateperone for five days, followed by a five-day washout period [19]. 

The primary outcome measures focused on evaluating the pharmacokinetics of lumateperone over seven weeks after administration, including parameters such as the maximum observed plasma concentration of lumateperone and its metabolites, time of maximum observed plasma concentration, area under the plasma concentration-time curve, and half-lives [19]. The secondary outcome measures assessed the occurrence of any TEAEs during the same period [19]. 

Although the study was completed in December 2022, the final results have not been released yet [19]. Once available, these results will provide valuable insights into the safety and tolerability of lumateperone as an injectable formulation for treating schizophrenia.

Ongoing trials on pediatric populations with schizophrenia

A separate ongoing clinical trial (NCT04779177) is underway to assess the safety and tolerability of lumateperone in pediatric populations aged 13-17 years who have been diagnosed with schizophrenia or schizoaffective disorder [20]. Similar to the previous trial, this study is a phase I open-label, non-randomized trial. It involved 26 participants who received either lumateperone 42 mg or lumateperone 28 mg for five days orally [20]. While the results of this trial have not been published yet, they are anticipated to provide valuable insights into the potential of lumateperone as a treatment option for pediatric patients with schizophrenia.

These ongoing trials underscore the continuous efforts to explore and expand the use of lumateperone in different populations and formulations. The results of these studies will help inform clinical practice and provide further insights into the potential benefits and safety of lumateperone in treating schizophrenia, particularly in the context of injectable administration and pediatric populations. The trial discussed above is summarized in Table 1.

**Table 1:** Results and comparison of clinical trials where lumateperone was used in patients with schizophrenia. PANSS: Positive and Negative Syndrome Scale; CGI-S: Clinical Global Impression-Severity; EPS: extrapyramidal symptoms.

Clinical trial	Number of participants	Length of study	Type of study	Endpoint measurements	Treatment arms	Results
Lieberman et al., 2015 [17]	335	Four weeks	Randomized, double-blind, placebo-controlled, and active-controlled	PANSS score	Lumateperone (42 mg and 84 mg), risperidone, placebo	-Lumateperone 42 mg showed greater antipsychotic efficacy compared to placebo, reducing positive symptoms significantly. -Lumateperone 84 mg reduced positive symptoms and general psychopathology but did not reach statistical significance compared to placebo. -Both doses of lumateperone were well tolerated with low discontinuation rates and adverse events. -Somnolence/sedation was the most commonly reported adverse event, with higher incidence rates for lumateperone 84 mg. -Neither dose of lumateperone was associated with EPS. -Lumateperone had a benign metabolic profile, with lower prolactin levels, fasting glucose, total cholesterol, and triglycerides compared to risperidone. -There was a tendency for less weight gain with lumateperone compared to risperidone, but the difference did not reach statistical significance.
Corell et al, 2020 [18]	450	Four weeks	Randomized, double-blind, placebo-controlled	PANSS score	Lumateperone 28mg/d, 42mg/d, placebo	-Administration of 42 mg of lumateperone resulted in a significant improvement in the PANSS total score compared to placebo (-4.2; p = 0.02). -Patients who received 28 mg of lumateperone showed a minor reduction in symptoms (-2.6; p = 0.16) compared to the 42 mg group but did not differ significantly from placebo.

Lumateperone for treating bipolar depression

The FDA has approved lumateperone for treating bipolar depression. Its approval in December 2019 was based on a series of clinical trials demonstrating its efficacy in improving depressive symptoms in patients with bipolar disorder [4]. In this section, we explore some notable trials that played a pivotal role in approving lumateperone for managing bipolar depression. These trials will provide insight into the drug's safety profile and effectiveness in treating bipolar depression.

Assessing the effectiveness and safety of lumateperone in six-week trials for the treatment of bipolar depression

A phase III randomized, double-blind, placebo-controlled study was conducted by Calabrese et al. [21] to determine the efficacy and safety of lumateperone in patients with bipolar I or bipolar II disorder experiencing a major depressive episode. The study involved 377 patients randomized in a 1:1 ratio to receive 42 mg/day of lumateperone or placebo, administered orally once daily in the evening for six weeks. The study's primary and critical secondary efficacy endpoints were the change from baseline to day 43 on the Montgomery-Åsberg Depression Rating Scale (MADRS) and the total score on the Clinical Global Impressions Scale-Bipolar Version Severity scale (CGI-BP-S), respectively [21].

The study results showed that patients who received lumateperone had significantly greater improvement in their MADRS score and CGI-BP-S total score compared with placebo on day 43 [21]. The MADRS superiority for lumateperone over placebo was observed in patients with both bipolar I and bipolar II disorders [21]. The TEAEs reported were somnolence and nausea, which occurred at a clinically greater rate with lumateperone than with a placebo. The incidence of extrapyramidal symptom-related TEAEs was statistically insignificant. Minimal changes were observed in weight, vital signs, metabolism, or endocrine function [21].

Calabrese et al. concluded that lumateperone at 42 mg/day significantly improved depression symptoms and was generally well tolerated in patients with depressive episodes associated with bipolar I and bipolar II disorders. Interestingly, the dosage of 42 mg/day was also found to be the optimal dosage for the management of schizophrenia [17,18]. These results suggest that lumateperone may be a promising adjunctive therapy for treating bipolar depression, with a favorable safety profile [21]. 

Lumateperone as an adjunct to lithium or valproate

Continuing on the findings by Calabrese, Suppes et al. [22] also aimed to investigate the efficacy and safety of lumateperone as an adjunctive therapy to lithium or valproate in patients with bipolar depression who had inadequate therapeutic responses to these mood stabilizers. The study was a phase 3, randomized, double-blind, placebo-controlled trial that enrolled 529 patients with bipolar I or II disorder experiencing a major depressive episode [22].

The patients were randomized to receive either lumateperone 28 mg (n = 176), lumateperone 42 mg (n = 177), or placebo (n = 176) for six weeks. The primary efficacy endpoint was the change in the MADRS total score. In contrast, the key secondary efficacy endpoint was the change in the CGI-BP-S depression subscore. Safety assessments included adverse events, laboratory evaluations, vital signs, EPS, and suicidality [22].

The results showed that patients treated with adjunctive lumateperone 42 mg had a significantly greater improvement in MADRS total score and CGI-BP-S depression subscore compared to those who received a placebo. Lumateperone 28 mg also showed significant improvement in MADRS total score and improvement in the CGI-BP-S depression subscore [22]. Furthermore, adjunctive lumateperone treatment was generally well tolerated, with TEAEs reported at rates <5%. The risk of EPS, metabolic abnormalities, or increased prolactin was minimal [22].

The study demonstrated that lumateperone adjunctive therapy significantly improved depression symptoms in patients with bipolar depression who had an inadequate therapeutic response to lithium or valproate. Lumateperone was generally well tolerated, with a low risk of adverse events, making it a promising treatment option for bipolar depression. However, further research is needed to confirm the long-term safety and efficacy of lumateperone in this population [22].

Lumateperone for acute bipolar depression

Durgam et al. [23] performed a fascinating study on the use of lumateperone in acute bipolar depression. The objective of this study was to assess the efficacy, safety, and tolerability of cariprazine in adult patients with acute bipolar I depression [23]. The study was an eight-week multinational, multicenter, randomized, double-blind, placebo-controlled, parallel-group, fixed-dose study. Patients were randomly assigned to receive placebo or cariprazine at 0.75, 1.5, or 3.0 mg/day. The primary and secondary efficacy parameters were the changes from baseline to week 6 on the MADRS and the CGI-S, respectively [23].

The results showed that cariprazine at 1.5 mg/day demonstrated consistent efficacy compared with placebo across outcomes and was generally well tolerated, suggesting its efficacy in treating bipolar I depression [23]. The most common adverse events in cariprazine-treated patients were akathisia and insomnia, and weight gain was slightly higher with cariprazine than with placebo. The 3.0 mg/day dosage also showed some efficacy but was insignificant when adjusted for multiple comparisons. The 0.75 mg/day dosage was similar to placebo [23]. This study found that cariprazine at 1.5 mg/day demonstrated consistent efficacy compared with placebo across outcomes. It was generally well tolerated, making it a potential treatment option for bipolar I depression [23]. The findings for these trials are summarized in Table 2.

**Table 2:** Results and comparison of clinical trials where lumateperone was used in BPDI and BPDII patients in the management of MDE. MADRS: Montgomery-Åsberg Depression Rating Scale; CGI-BP-S:  Clinical Global Impressions Scale-Bipolar Version severity scale; BPDI bipolar disorder type I; BPDII: bipolar disorder type II; MDE: major depressive episodes; TEAEs: treatment-emergent adverse events.

Clinical trial	Number of participants	Length of study	Type of study	Endpoint measurements	Treatment arms	Results
Calabrese et al., 2021 [21]	381	Six weeks	Multicenter-multinational randomized, double-blind, placebo-controlled study in 1:1 ratio	MADRS SGI-BP-S	Lumateperone 42 mg/day, placebo	-Patients who received lumateperone showed significantly greater improvement in MADRS score and CGI-BP-S total score compared to placebo at day 43. -Lumateperone was effective in patients with both bipolar I and bipolar II disorders. -The most commonly reported TEAEs were somnolence and nausea, occurring more frequently with lumateperone than with a placebo. -The incidence of extrapyramidal symptom-related TEAEs was statistically insignificant. Minimal changes were observed in weight, vital signs, metabolism, or endocrine function. -The 42 mg/day dosage of lumateperone was found to be effective for managing both schizophrenia and bipolar depression. -The study suggests that lumateperone may be a promising adjunctive therapy for treating bipolar depression with a favorable safety profile.
Suppes et al., 2023 [22]	529	Six weeks	Randomized, double-blind, placebo-controlled study in a 1:1:1 ratio	MADRS SGI-BP-S	Lumateperone 28 mg/day, 42 mg/day, placebo (+ lithium or valproate)	-Patients treated with adjunctive lumateperone 42 mg had a significantly greater improvement in MADRS total score and CGI-BP-S depression subscore compared to placebo. -Lumateperone 28 mg also showed significant improvement in MADRS total score and CGI-BP-S depression subscore. -Adjunctive lumateperone treatment was generally well tolerated, with somnolence, dizziness, and nausea being the most commonly reported adverse events. -The risk of extrapyramidal symptoms, metabolic abnormalities, or increased prolactin was minimal. -Lumateperone adjunctive therapy significantly improved depression symptoms in patients who had an inadequate therapeutic response to lithium or valproate. -Lumateperone was well tolerated with a low risk of adverse events, suggesting it as a promising treatment option for bipolar depression.
Durgam et al, 2015 [23]	571	Eight weeks	Randomized, double-blind, placebo-controlled, parallel-group, fixed-dose study	MADRS SGI-BP-S	Cariprazine -0.75 mg/day, 1.5 mg/day, 3.0 mg/day, placebo	-Cariprazine at 1.5 mg/day demonstrated consistent efficacy compared to placebo on both MADRS and CGI-S. -Cariprazine at 1.5 mg/day was generally well tolerated. The most common adverse events in cariprazine-treated patients were akathisia and insomnia. -Weight gain was slightly higher with cariprazine than with placebo. -The 3.0 mg/day dosage showed some efficacy but was not statistically significant when adjusted for multiple comparisons. -The 0.75 mg/day dosage was similar to placebo. Cariprazine at 1.5 mg/day demonstrated consistent efficacy compared to placebo, suggesting it is a potential treatment option for bipolar I depression.

Lumateperone may impact mood through mediating inflammatory pathways

In their research, Dutheil et al. aimed to investigate the potential mechanisms through which lumateperone can effectively manage mood disorders by modulating inflammatory pathways [24]. Highlighting the connection between the immune system and psychiatric illness, the researchers showed that lumateperone might alleviate symptoms of depression by normalizing pathological inflammation [24,25].

The study involved male and female C57BL/6 mice subjected to an acute immune challenge. Lumateperone demonstrated its ability to reduce elevated levels of the key proinflammatory cytokines, including IL-1β, IL-6, and TNF-α, in both the brain and serum [24,26]. Moreover, in male mice experiencing acute behavioral stress, lumateperone also effectively decreased proinflammatory cytokines [24]. Furthermore, the researchers also observed that lumateperone influenced pathways involved in maintaining tissue integrity and supporting blood-brain barrier function. For instance, lumateperone altered the expression of claudin-5 and intercellular adhesion molecule 1 [24,27-29].

Additionally, in acutely stressed male Sprague-Dawley rats, lumateperone demonstrated anxiolytic and antianhedonic effects while enhancing activity in the mammalian target of the rapamycin complex one pathway in the prefrontal cortex [24,30]. The mammalian target of rapamycin complex 1 (mTORC1) is a key signaling pathway involved in various cellular processes, including inflammation. Activation of mTORC1 has been linked to promoting proinflammatory responses [30]. When mTORC1 is activated, it stimulates the production of proinflammatory cytokines and chemokines. These molecules play crucial roles in initiating and propagating inflammation within the body [30]. Taken together, the researchers' preclinical findings indicate that lumateperone, in addition to its modulation of multiple neurotransmitter systems, may also act by mitigating the impact of acute inflammatory processes [24].

Limitations and shortcomings of lumateperone

While the results of the aforementioned clinical trials show promising outcomes, certain limitations within this study should be considered when interpreting these findings. The first limitation pertains to the limited amount of prior research on lumateperone in clinical settings. Due to its recent introduction into the market, available studies are scarce compared to other medications used to treat schizophrenia and bipolar depression. The lack of comprehensive research could hinder the evaluation of lumateperone's true efficacy and safety profile, particularly when comparing it to other established treatments for these conditions. The studies selected for this paper only compared lumateperone to either a placebo or a single alternative drug (such as risperidone or cariprazine), thereby restricting the ability to draw significant conclusions regarding the comparative benefits or drawbacks of lumateperone as a treatment option, especially when trying to determine if it should be considered a first-line treatment option. However, this limitation will gradually diminish as lumateperone becomes more widely used and additional research becomes available. 

The second limitation is related to time constraints. The average duration of patient follow-up in the clinical trials used for this study was approximately 5.8 weeks. Although this duration was sufficient to demonstrate an acceptable short-term safety profile, further research is required to assess the long-term efficacy and safety of lumateperone. Future studies must allow for longer periods of follow-up to study and report on the drug's sustained efficacy, tolerability, and potential adverse events over extended treatment periods before making more definitive statements about its long-term effects. 

While some other minor limitations could be discussed, such as potential publication bias, a narrow selection profile of sample participants (such as age range and comorbidities), and potential biases and blinding challenges that commonly occur across randomized control trials, there is little reason to believe these potential limitations would have a significant impact on the interpretation of this study when compared to the two more major limitations described above. Ultimately, given how relatively new lumateperone is to the market of psychotropic drugs, it is essential to await further research and evidence regarding the effectiveness, tolerability, and long-term outcomes of lumateperone in treating schizophrenia and bipolar depression. Once the safety and efficacy of lumateperone are established, we need more head-to-head drug trials to compare with our current standards of treatment.

## Conclusions

In conclusion, we have highlighted the potential of lumateperone, marketed as Caplyta, to be an effective psychotropic medication for treating schizophrenia and bipolar depression. Following its FDA approval in 2019 for treating schizophrenia and later in 2021 for bipolar depression, lumateperone has become a promising option for those with these afflictions. This review began with lumateperone's mechanism of action, discussing its unique effects on neuromodulatory receptors/transporters such as dopamine D2, DAT, NMDA receptor, and SERT. Likely, its partial agonist properties, along with the antagonism of the DAT, play a role in its low risk for EPS. The distinctive pharmacokinetics of lumateperone were also examined and discussed.

A notable finding of lumateperone is its favorable metabolic profile, likely secondary to its lack of affinity for histamine and muscarinic receptors. As discussed, the CATIE trials exposed the SGA's tendency to cause MetS. Outperforming other SGAs, lumateperone demonstrates positive outcomes regarding mean body weight, BMI, and waist circumference. Furthermore, the efficacy and safety of lumateperone in managing schizophrenia and bipolar depression have been highlighted in numerous trials discussed in this review. Through extensive analysis, compelling evidence has showcased lumateperone's advantages over the existing antipsychotic arsenal. The need for further research to uncover additional aspects, hidden side effects, and potential drawbacks should not be taken lightly. Nonetheless, the promising benefits of lumateperone and its potential for advancing the treatment of mental illness should be cause for excitement. In summary, this drug review has illuminated the potential of lumateperone, highlighting its efficacy, safety, favorable metabolic profile, and success in clinical trials. We call for continued research and drug trials to fully understand the limitations of lumateperone. Lumateperone is a beacon of hope, offering new possibilities for patients and clinicians.
